# Ritueller sexueller Missbrauch: Orientierung am Patientenwohl in einer polarisierten Debatte

**DOI:** 10.1007/s00115-024-01652-2

**Published:** 2024-04-17

**Authors:** Jörg M. Fegert, Frank Urbaniok

**Affiliations:** 1 Klinik für Kinder- und Jugendpsychiatrie/Psychotherapie, Universitätsklinikum Ulm, Steinhövelstraße 5, 89075 Ulm, Deutschland; 2 Fachbereich Psychologie, Universität Konstanz, Konstanz, Deutschland 78457

**Keywords:** Behandlungsfehler, Verschwörungserzählung, Glaubhaftigkeitsbegutachtung, False Memories, Dissoziative Identitätsstörung (DIS), Medical treatment error, Conspiracy narrative, Credibility assessment, False memories, Dissociative identity disorder

## Abstract

**Hintergrund:**

Hintergrund des Beitrags bildet die polarisierte mediale Debatte um rituelle sexuelle Gewalt gegen Kinder. Es haben sich zwei Lager gebildet, die sich gegenüberstehen und denen ein sachlicher Austausch miteinander nicht möglich ist.

**Ziel:**

Der Artikel verfolgt daher das Ziel, einen klinischen Beitrag zur Überbrückung der Differenzen zu leisten und argumentiert vor allem im Sinne des Wohls von Patient:innen dafür, sich auf eine Evidenzbasierung sowohl in der Krankenbehandlung als auch im wissenschaftlichen Diskurs zu besinnen.

**Material und Methoden:**

Der Beitrag basiert auf der kritischen Auseinandersetzung mit exemplarischer Fachliteratur, öffentlichen Untersuchungsberichten zu Fehlbehandlungen vor allem in der Schweiz sowie auf die Medienberichterstattung.

**Ergebnisse:**

In bestimmten psychotraumatologischen Kreisen sowie in den Medien (gerade auch in sozialen Medien auf Plattformen wie Telegram) ist ein Verschwörungsnarrativ über ein großes Täter:innennetzwerk, welches im rituellen Kontext Kindern schwerste Gewalt antue, präsent. Ein unkritischer Glaube z. B. an Konzepte wie „mind control“ (Vorstellung eines absichtlichen und planvollen Herbeiführens einer dissoziativen Identitätsstörung) hat zu Fehlbehandlungen von Patient:innen sowie im Gegenzug zu einem grundlegenden Misstrauen gegenüber deren Aussagen geführt. Dadurch droht diesen ohnehin vulnerablen Patient:innen weiterer Schaden, was den basalen Prinzipien medizinischer Ethik widerspricht.

## Abstract (en)

**Background:**

The background to this article is a polarized media debate about ritual violence against children in German speaking countries. Two opposing positions have formed, which are unable to engage in a reasonable exchange with each other.

**Objective:**

The article therefore aims to contribute to bridging the differences and argues, above all in the interests of patient well-being, for positioning oneself beyond polarization and to reflect on an evidence-base in treatment as well as in scientific discourse.

**Material and methods:**

The article is based on a critical examination of exemplary scientific literature, public investigation reports and diverse media coverage.

**Results:**

In certain psychotraumatology circles and in the media (especially on social media, such as telegram), there is a conspiracy narrative about a large network of perpetrators who inflict the most serious sexual violence on children in a ritual context. An uncritical belief in this has already led to mistreatment of patients and also to a fundamental mistrust of their statements. This threatens these already vulnerable patients with further harm, which contradicts the basic principles of medical ethics.

## Öffentliche Zuspitzung der Debatte um rituellen Missbrauch

Medienberichterstattung, eine Satiresendung im deutschen Fernsehen und diverse Reaktionen in der Fachwelt haben in Deutschland und der Schweiz zu einer Polarisierung der Debatte um rituellen Missbrauch geführt. Solche Lagerbildungen und vorschnellen Zuordnungen verhindern die notwendige fachliche Debatte und machen gleichzeitig aus einzelnen Aspekten im Opferschutz fast schon „Glaubenskriege“. Das schadet allen.

Der fachliche Austausch und ethische Grundprinzipien in der Krankenbehandlung werden beschädigt und die epistemische testimoniale Ungerechtigkeit im Umgang mit Aussagen von Betroffenen, insbesondere wenn sie sich aufgrund psychischer Probleme in Behandlung begeben haben, wird verschärft (Tab. 1).

**Tab. 1:** Prinzipien der heilberuflichen Ethik (Belmont-Principles) als Kriterien für das Patientenwohl

Prinzip		Bedeutung
Respekt vor der Selbstbestimmung	Respect for persons/autonomy	Krankenbehandlung sollte Unabhängigkeit der Patient:innen fördern, kleinstmögliche Eingriffe in persönliche Freiheiten und Grundrechte
Nichtschadensgebot	Non-maleficience	Krankenbehandlung sollte den Zustand von Patient:innen nicht verschlechtern
Handeln zum Wohl der Patient:innen	Beneficience	Krankenbehandlung sollte Patient:innen nutzen und eine Verbesserung der Symptomatik mit sich bringen
Gerechtigkeit	Justice	Krankenbehandlung sollte rechtebasiert sein, nicht die Rechte anderer verletzten sowie rechtskonforme Zugänge zu Hilfen bzw. sozialer Entschädigung schaffen

[16, 22]

Zweifel an Aussagen, welche in sehr speziellen Therapiesettings aufgetreten sind, werden generalisiert und die Inanspruchnahme von Krankenbehandlung unberechtigterweise generell problematisiert. In Deutschland ist soeben das neue soziale Entschädigungsrecht nach dem SGB XIV in Kraft getreten. Es bringt teilweise wichtige, hart erkämpfte Fortschritte für Betroffene (z. B. Traumaambulanzen und Beweiserleichterungen im Verfahren). Kurz vor dem Inkrafttreten relativierte der Gesetzgeber die Grundannahme, dass eine Begutachtung zur Verifizierung der Tat im Sozialrecht die Ausnahme sein soll, durch eine Veränderung der Versorgungsmedizinverordnung genau bei Patient:innen mit psychischen Gesundheitsstörungen. Da sie sich in Psychotherapie befinden, wird eher von der Notwendigkeit der Begutachtung ausgegangen. Diese Zweifel schaden den Betroffenen und verhindern die notwendige Inanspruchnahme zeitnaher Krankenbehandlung.

Menschen mit psychischen Gesundheitsstörungen werden grundlegend infrage gestellt, sodass ihr Stigma verstärkt und die Inanspruchnahme von Hilfe ebenfalls stigmatisiert wird. Wenn auf der anderen Seite ohne Einschränkung gesagt wird, dass man aus Prinzip Aussagen von Betroffenen nicht hinterfrage und auf jede Realitätsprüfung verzichten müsse, ist das therapeutisch nicht sinnvoll und verhindert, frühzeitig Opfer von Falschbeschuldigungen zu erkennen. Frühe Kindheitsbelastungen – darunter sexualisierte Gewalt gegen Kinder – sind häufig und spielen in vielen Anamnesen eine Rolle [28, 31]. Es geht deshalb nicht darum, biographische Erinnerungen auch an lang zurückliegende Ereignisse oder gar die Glaubwürdigkeit von Betroffenen als Gruppe generell infrage zu stellen. Vielmehr geht es um die realitätsorientierte Beurteilung der Glaubhaftigkeit in bestimmten, begründeten Einzelfällen. Dabei kann im Opferhilfekontext und in der Krankenbehandlung auch nicht die vom deutschen BGH in Strafsachen zum Grundprinzip stilisierte Annahme, dass zunächst davon auszugehen sei, dass die Aussage von Betroffenen falsch sei („Nullhypothese“ sensu BGH), allgemein handlungsleitend sein. Es gibt viele gute Gründe, Erlebtes nicht zu offenbaren.

Der französische Facharzt und Resilienzforscher Boris Cyrulnik beschreibt in seiner Autobiographie „Sauve-toi, la vie t’appelle“ („Rette Dich, das Leben ruft“) seine Flucht als kleiner Junge vor einer Razzia aus der Synagoge in Bordeaux. Er beschreibt, wie er nach einigen Versuchen nach dem Krieg aufgehört hat, darüber zu sprechen, weil die Gesellschaft in Frankreich bis in die 1970er-Jahre nicht hören und nicht wahrhaben wollte, dass es auch französische Kollaborateure gab, dass Naziverbrechen auch in Frankreich stattgefunden hatten und dass nicht alle in der Résistance waren. Er beschreibt aber auch als erfahrener Neuropsychiater, wie sich seine Erinnerungen aufgrund der emotionalen Verarbeitung, die ihm das Überleben ermöglicht hat, teilweise in seinen späteren Narrativen verändert haben. Er überprüft dies mithilfe von Aussagen von Personen, die ihm beim Überleben geholfen haben und macht hier gedächtnispsychologisch sehr deutlich, wie das Kerngeschehen für ihn immer erinnerlich und abrufbar war, wie aber Details, entsprechend seiner emotionalen Verarbeitung des Erlebten, teilweise seine Erinnerung überformt haben. Insofern ist die Inkonsistenz einzelner Aussagedetails, die oft im strafrechtlichen Kontext überbewertet wird, kein Grund für berechtigte Zweifel am erlebten Kerngeschehen [3, 4].

Boris Cyrulnik ist auch ein Beispiel dafür, dass eben dieses Kerngeschehen nie völlig aus der Erinnerung getilgt war und erst zu einem viel späteren Zeitpunkt aus dem Nichts aufgetaucht wäre, ohne an irgendeine Spur der eigenen Erinnerungskontinuität anzuknüpfen. Diese Präsenz und Kontinuität einer Kernerinnerung entspricht auch dem Befund, dass Erlebnisse, die mit hoher emotionaler Intensität verbunden sind, mit höherer und nicht geringerer Wahrscheinlichkeit erinnert werden [7] Dementsprechend sind sich ungewollt aufdrängende Erinnerungen (Flashbacks) ein wesentliches Symptom von Traumafolgestörungen [9].

## Positionierung jenseits der Polarisierung

Als Experten, die in der aktuellen Debatte um das schlecht definierte Phänomen ritueller Missbrauch möglicherweise unterschiedlichen Lagern zugeordnet werden, wollen wir uns vor dem Hintergrund unserer forensischen, psychotherapeutischen und wissenschaftlichen Erfahrung gemeinsam positionieren, um eine differenzierte Einschätzung jenseits dieser polarisierten Debatte zu geben. Dies ist uns wichtig, weil die Aufspaltung in Lager nicht weiterführt. Im Gegensatz zur wissenschaftlichen Erforschung der Folgen von Traumata, im Gegensatz zu evidenzbasierten, in Studien vielfach replizierten und gut untersuchten therapeutischen Methoden, hat sich im Bereich sog. ritueller Gewalt und in der sog. „False-memory-Debatte“ seit 30 Jahren wissenschaftlich kaum etwas getan (für einen Überblick: [12]). Dies ist angesichts der fulminant angestiegenen Forschungsliteratur zu sexualisierter Gewalt gegen Kinder und Jugendliche bemerkenswert.

Die Diskussion um organisierte sexualisierte und rituelle Gewalt wurde und wird begleitet von Zweifeln an den Schilderungen und vom Hinterfragen der Existenz, insbesondere von ritueller Gewalt. Während die dringend notwendige gesellschaftliche Diskussion um zuletzt medial bekannt gewordene Fälle organisierten Kindesmissbrauchs durch Netzwerke von Täter:innen, inklusive der Herstellung und Verbreitung von Missbrauchsabbildungen (z. B. um das Pädosexuellennetzwerk „Elysium“), gesellschaftlich und wissenschaftlich weitestgehend ausbleibt, kann bezüglich des Themas rituelle Gewalt in Internetforen und Fachkreisen eine hoch emotional geführte Diskussion und eine extreme Polarisierung beobachtet werden. Dabei werden auch Beratungsangebote der UBSKM infrage gestellt und Projekte des BMSFJ angegriffen (z. B. [13] mit einer guten Übersicht der eingeschränkten Literaturlage). Einerseits gibt es Menschen, die in unterschiedlichen Kontexten von erlebter Gewalt im rituellen Kontext berichten, sowie Fachkräfte, die nach eigenen Angaben mit Betroffenen gearbeitet haben. Andererseits wird die Position vertreten, dass geschilderte Erfahrungen als potenzielle Produkte eines induzierten Verschwörungsnarrativs im Sinne von „false memories“ angesehen werden müssen, die durch eine intensive Befassung mit der Thematik sowie durch den Austausch mit anderen mutmaßlich Betroffenen entstünden oder auch (bewusst oder unbewusst) von Therapeut:innen induziert würden. Dabei steht gerade nicht die Existenz schwerer sexualisierter Gewalt, organisierter Täterschaften oder traumatisierender Erfahrungen infrage, sondern v. a. spezifische Aspekte wie etwa das sog. „mind control“ und bestimmte repetitiv präsentierte Inhalte, wie die Tötung von Babys und das angebliche Trinken von Kinderblut oder aus rein ideologischen Gründen praktizierte schwerste Misshandlungen.

Mittlerweile in der Schweiz aufgedeckte Fälle zeigen, dass Epigonen eines „eingeweihten“ Kreises von Therapeut:innen im Rahmen vermeintlich festgestellter ritueller Gewalt Pseudoerinnerungen oder -narrative suggeriert haben [18, 21]. Dieses Fehlverhalten in der Krankenbehandlung hat standesrechtliche, zivilrechtliche und ggf. auch strafrechtliche Bedeutung und ruft damit die forensische Psychiatrie zu Recht auf den Plan.

Es gilt, Phänomene und Motive klar zu analysieren, Tatkontexte und begünstigende Faktoren zu verstehen und damit gleichzeitig zu versuchen, einen Beitrag dazu zu leisten, andere hilfesuchende Personen vor Schäden durch eine Fehlbehandlung zu bewahren und die oft dramatischen Folgen, von zum Teil erfolgenden Falschbeschuldigungen von Drittpersonen, zu verhindern. In jüngster Zeit wird ausgehend von den Schweizer Fällen die Problematik falscher Erinnerungen, insbesondere im Zusammenhang mit therapeutischen Fehlbehandlungen, übergeneralisierend thematisiert, was ebenfalls Anlass zur Sorge ist.

Dabei besteht die Gefahr, dass die fachliche Diskussion und die mit ihr einhergehende notwendige Differenzierung in einer vielschichtigen Thematik durch eine teils reflexhaft einsetzende und durch eine starke Emotionalität geprägte Polarisierung versperrt wird. So gilt es zunächst, folgenden Grundsatz festzuhalten: Es gibt organisierte, grausamste Formen sexuellen Missbrauchs. Viele Betroffene machen die Erfahrung, dass ihnen nicht geglaubt wird, und sie haben größte Schwierigkeiten, zu ihrem Recht zu kommen sowie angemessene Unterstützung und Behandlung zu erhalten. Vor diesem Hintergrund wäre es fatal, wenn die Diskussion zu „false memories“ und Falschbeschuldigungen diese belastende Situation weiter verschärfen würde, indem Aussagen von Opfern generell in Zweifel gezogen und diskreditiert würden. Umgekehrt wäre es genauso falsch, wenn aufgrund dieser Befürchtung die Diskussion zu „false memories“ und Falschbeschuldigungen als Angriff auf die Rechte tatsächlicher Opfer verstanden und aus diesem Grund die notwendige fachliche Diskussion blockiert würde.

Betroffene sexuellen Missbrauchs sind zweifellos Personen, denen vertrauensvoll begegnet werden sollte und die ein Recht auf Schutz und Unterstützung haben. In gleicher Weise sind Menschen, die von Falschbeschuldigungen betroffen sind, ebenfalls Opfer. Es gilt in jedem Fall zu verhindern, dass beide Opfergruppen in einer ideologisch polarisierten Diskussion gegeneinander ausgespielt werden (Sowohl-als-auch statt Entweder-oder).

Manche Opfervertreter:innen und Traumatherapeut:innen wandten sich zunächst vehement gegen die öffentliche Berichterstattung und die fachlichen Kritiken zu solchen Traumatherapien. Aber nicht die Aufdeckung von Fehlbehandlungen, „false memories“ und Falschbeschuldigungen stellen eine Gefahr für die Glaubhaftigkeit der Aussagen tatsächlicher Opfer, Unterstützungsangebote und Traumatherapien dar, sondern, im Gegenteil, der Versuch, diese Phänomene zu bagatellisieren und zu tabuisieren. Denn eine fehlende Aufarbeitung hat das Potenzial, über Jahrzehnte erzielte Fortschritte in Bezug auf wirksame, evidenzbasierte Therapieangebote, Beratung und Unterstützung von Betroffenen sexuellen Missbrauchs zu beschädigen und generell zu diskreditieren. Darum sind eine sachliche Darstellung und Aufarbeitung von Falschbeschuldigungen und suggestivem Vorgehen in Therapien keine Gefahr für die wichtige Arbeit mit der Vielzahl tatsächlicher Opfer sexuellen Missbrauchs, sondern sie ist wichtig, um generelle Vorbehalte gegenüber Aussagen von Opfern und gegenüber der Arbeit von Opferberater:innen und Traumatherapeut:innen zu verhindern.

## Charakteristika der in der Schweiz aufgearbeiteten Fälle

Anhand der bislang aufgearbeiteten Fälle in der Schweiz im Zusammenhang mit angeblicher ritueller Gewalt zeigten sich typische Elemente:

### Verschwörungserzählung

Teilweise wird von Therapeut:innen eine QAnon-ähnliche Ideologie vertreten. Demnach soll eine geheime, im Untergrund arbeitende (satanische oder anders weltanschaulich geprägte) sektenähnliche Organisation aus ideologischen Gründen systematisch Kinder quälen, missbrauchen und töten. Es gibt trotz zahlreicher Ermittlungsverfahren bislang keinen einzigen Fall, in dem sexuelle Missbrauchshandlungen aus ideologischen Motiven durch eine mächtige subversiv arbeitende Organisation nachgewiesen wären. Die fehlenden Belege werden in einer für Verschwörungserzählungen typischen Argumentationskette als Argument für die Macht dieser Täter:innenorganisationen angeführt und damit gegen faktenbezogene Einwände immunisiert. Damit entsteht ein geschlossenes Verschwörungsnarrativ, das sich aus sich selbst heraus begründet^1^.

Aus einem wissenschaftstheoretischen Ansatz heraus ist in diesem Zusammenhang auf das Prinzip der Falsifizierbarkeit zu verweisen, welches wissenschaftliche Thesen von Glaubensüberzeugungen unterscheidet. Es ist typisch für Verschwörungserzählungen, dass das Kriterium der Falsifizierbarkeit außer Kraft gesetzt wird. Dies gilt auch für die Debatte um rituelle Gewalt: Das Fehlen jeglicher Belege wird nicht als Falsifikationskriterium akzeptiert. Vielmehr wird es als positive Bestätigung des Narrativs interpretiert, indem dies die Macht der geheimen Organisation belege. In für Verschwörungsnarrative typischer Weise wird damit jegliche Falsifikationsmöglichkeit eliminiert und das Narrativ gegen Fakten immunisiert, die es infrage stellen könnten [17].

In diesem Verschwörungsnarrativ spielen die Diagnose der – zum Teil angeblich bewusst induzierten – DIS und die Theorie über ein aufgrund von Spezialwissen erzeugtes „mind control“ eine wichtige Rolle.

Personen, die auf diese Problematik hinweisen, werden in der Regel als Angehörige oder Unterstützer:innen eines vermuteten geheimen Täter:innennetzes angesehen.

### Dissoziative Identitätsstörung

Als Diagnosekategorie ist die dissoziative Identitätsstörung (DIS) im DSM und zukünftig in der ICD-11 aufgeführt. In der ICD-11 ist die Diagnose 6B64 sehr restriktiv definiert. Sie erfordert neben symptomatischen und klinischen Grundkriterien insbesondere die Berücksichtigung einer breiten Palette von Ein- und Ausschlusskriterien, welche stringente differenzialdiagnostische Überlegungen erzwingen und eine leichtfertige Diagnosestellung verhindern soll. Diese Vorgaben sind in der englischen Originalversion der ICD-11 im Detail^2^ beschrieben, noch nicht jedoch in der provisorischen deutschen Übersetzung.

Unabhängig von einer generellen konzeptionellen Skepsis in Bezug auf die Diagnose DIS ist zu vermuten, dass aufgrund von Unkenntnis dieser diagnostischen Einschränkungen, diese Diagnose in bestimmten therapeutischen Subkulturen überdiagnostiziert wird. In letzter Zeit mag auffallen, dass die neue DSM-5-Diagnose der DIS in Foren und teilweise auch in Fachdiskursen die ICD-10-Diagnose „multiple Persönlichkeitsstörung“ abgelöst hat. Die Kurzfassung DIS als Label wird auch als Kennwort verwendet und erfreut sich in einschlägigen Foren, auch bei manchen Jugendlichen, besonderer Beliebtheit [26]. Ähnliche Effekte sind bei anderen, erheblich geänderten Definitionen psychischer Störungen durch die ICD-11 nicht bemerkbar. Für die DIS gibt es derzeit keine leitlinienbasierte Therapie. In den beschriebenen Fällen fiel auf, dass im Laufe der Behandlung bei den Patient:innen immer weitere Persönlichkeiten hinzugekommen sind und therapeutisch erkennbar keine Versuche unternommen wurden, eine Reintegration zu funktionierenden integrierten Ich- und Selbstfunktionen anzustreben^3^. Unter erheblichem medialem Druck haben sich Einrichtungen und auch therapeutische Verbände in der Schweiz mittlerweile von einem solchen Vorgehen distanziert.

Im Zusammenhang mit Fehlbehandlungen, „false memories“ und Falschbeschuldigungen ist die DIS ein Einfallstor für Suggestion. Denn Patient:innen mit DIS werden nicht mehr als autonom handelnde und sich erinnernde Subjekte wahrgenommen. Vielmehr existieren gemäß dieser Theorie unbewusste, abgespaltene Persönlichkeitsanteile, die ein Eigenleben führen. Ihnen wurden in den vorliegenden Fällen von Therapeut:innen Bedeutungen, Funktionen und bestimmte Erinnerungen zugeschrieben, die durch die sog. Alltagspersönlichkeit nicht überprüft und entkräftet werden konnten, weil sie gemäß der Theorie gar keinen Zugang zu den Funktionen und Erinnerungen der abgespaltenen Persönlichkeitsanteile hat. Damit ist die DIS prädestiniert, als Eintrittspforte für die therapeutische Induzierung von „false memories“ zu dienen.

### „Mind control“

Sogenanntes „mind control“ wird in diesem Zusammenhang als die gezielte, durch Spezialwissen bestimmter Täter:innenkreise ermöglichte, Schaffung abgespaltener Persönlichkeitsanteile verstanden. Da diese Persönlichkeitsanteile durch die Täter:innen kreiert würden, könnten diese zeitlebens, ähnlich einer Fernsteuerung, Zugriff auf die Gesamtperson ausüben – ohne dass die betroffene Person davon Kenntnis hat (z. B. [10, 15]). Für dieses Konzept des sog. „mind control“ gibt es weder irgendeinen realen Beleg noch ist die ihr zugrunde liegende spekulative Annahme plausibel [27].

Im Frühjahr 2022 haben Behandelnde in Traumatherapiestationen in der Schweiz bei Patient:innen systematisch (angebliche) Erinnerungen an rituelle Gewalt induziert. Meistens wurde die in diesem Zusammenhang angenommene DIS durch Aufspaltung in immer weitere Unterpersönlichkeiten verstärkt bzw. chronifiziert. In Reportagen des Schweizer Fernsehens (SRF) wurden diverse weitere Fälle dargestellt, in denen die Erinnerungen offensichtlich nicht der Wahrheit entsprechen können. Vielmehr handelte es sich in den beschriebenen Fällen um ein Verschwörungsnarrativ, da die Phänomene des sog. „mind control“ und der absichtlich induzierten Abspaltung von Persönlichkeiten nicht belegbar sind. Mit dem Rückschluss von der Diagnose DIS auf die Gewalt lag zudem oft ein unüberprüfbarer Zirkelschluss vor. Mittlerweile wurde neben den Gutachten auch ein öffentlich zugänglicher Untersuchungsbericht vorgelegt, in dem Behandlungsfehler festgestellt wurden. Auf Grundlage des Berichts wurden zudem personelle Konsequenzen gezogen und Auflagen seitens der Aufsichtsbehörde gegenüber der betroffenen Klinik gemacht [18]. Die Autorenschaft kommt zu dem Ergebnis, dass einige Fachkräfte der untersuchten Traumatherapiestationen an die Verschwörungserzählung „rituelle Gewalt/mind control“ glaubten und dies, zumindest zu Teilen, in die Behandlung der Patient:innen eingeflossen ist. In den folgenden Reportagen wurden weitere ähnliche Fälle dargestellt, von denen zwei weitere Kliniken in der Schweiz betroffen sind. Zu einigen Fällen liegen mittlerweile auch Gerichtsentscheide im Zusammenhang mit, aus entsprechenden Erinnerungen resultierenden, Falschbeschuldigungen vor^4^.

In den Schweizer Fällen lassen sich die drei hier dargestellten Elemente in unterschiedlicher Kombination finden. Es ist allerdings darauf hinzuweisen, dass die Induzierung von „false memories“ und Falschbeschuldigungen auch unabhängig von diesen spezifischen, im Zusammenhang mit ritueller Gewalt vertretenen Überzeugungen auftreten können. Dann handelt es sich um durch Therapeut:innen ohne diesen spezifischen Hintergrund induzierte bzw. verstärkte „false memories“ aufgrund allgemeiner Suggestionen im Rahmen spekulativ angenommener tieferliegender Traumata z. B. bei Patient:innen, die über eine reale Traumatisierung berichten.

## Identifizierung von Falschbeschuldigungen und Glaubhaftigkeitsbegutachtung

Von manchen Vertreter:innen der Aussagepsychologie wird die Meinung propagiert, dass allein entsprechend der Rechtsprechung des deutschen BGH in Strafsachen sog. „wissenschaftliche Methoden“ in der Lage seien, Falschbeschuldigungen erkennen zu können. Darunter wird die im deutschsprachigen Raum übliche Methodik der Glaubhaftigkeitsbegutachtung verstanden, welche eine Reihe einzelner Hypothesen qualitativ in Bezug auf Aussagen „überprüft“ und zunächst davon ausgeht, dass die Aussage Betroffener unwahr ist [30]. Eine Methode, welche offensichtlich nicht wie ein diagnostischer Test geeignet ist, Angaben zur relativen Wahrscheinlichkeit zu machen, muss vor dem Hintergrund spezifischer Fragestellungen angewandt werden. Im strafrechtlichen Kontext ist eine Gewichtung zugunsten des Zweifelsgrundsatzes nachvollziehbar, um unbegründete Falschbeschuldigungen (falsch-positive) zu vermeiden. Dafür wird eine erhebliche Zahl falsch-negativer in Kauf genommen. Von einer aussagepsychologischen Begutachtung ist deshalb zu erwarten, dass konkrete Kriterien – von Plausibilitätsüberlegungen bis hin zu identifizierten Widersprüchen – zu einer Annahme führen müssen, dass die Erlebnisbasierung einer Aussage in Zweifel gezogen werden könnte. Hinzuweisen ist auch darauf, dass die Beurteilung der Glaubhaftigkeit von Aussagen und die Bewertung entsprechender Sachverhalte im Kernbereich juristischer Sachverhaltsbewertungen liegen.

Allerdings kann eine Erlebnisbasierung z. B. im sozialrechtlichen Kontext als hinreichend plausibel oder als eine mit überwiegender Wahrscheinlichkeit anzunehmende Schlussfolgerung angegeben werden, ähnlich wie bei einer Risikobeurteilung in einem forensisch-psychiatrischen Gutachten eine durch nachvollziehbar dargelegte Kriterien und Überlegungen (Deliktmechanismus) begründete Ausprägung des Rückfallrisikos bestimmt wird.

In diesem Zusammenhang ist aber auch darauf hinzuweisen, dass ähnlich wie bei anderen Gutachten, nicht eine Methode vorliegt, die zu richtigen Ergebnissen führt. Vielmehr garantieren vor allem qualifizierte und klinisch erfahrene Gutachter:innen, zu schlüssig begründeten und damit nachvollziehbar abgestützten Beurteilungen zu kommen, nicht eine davon unabhängige, angeblich wissenschaftlich begründete Methode.

## Forschungslücke hinsichtlich ritueller Gewalt

Bei der Sichtung der vorliegenden Literatur fällt einerseits auf, dass viele Studien auf selektiven Samples betroffener Patient:innen und Fachkräfte basieren [1, 23, 24], und andererseits, dass sich die Verbreitung von „Fachwissen“ im Bereich ritueller Gewalt bisher auf einen engen Kreis von Therapeut:innen beschränkt, welche hauptsächlich nicht datenbasiert vorgehen. Damit divergiert die Forschung hinsichtlich ritueller Gewalt von allen anderen Bereichen der Gewaltforschung. Demgegenüber gibt es diverse experimentelle Untersuchungen, die zeigen, dass „false memories“ induziert werden können [2, 20, 25]. Hahn [14], Dessecker [5] und Urbaniok [29] konstatieren, dass Belege für systematisch organisierten rituellen Missbrauch aus ideologischen Gründen fehlen und beschreiben eine problematische Induzierung von Narrativen durch Fachkräfte. Hahn [14] betont die Diskrepanz zwischen der hohen Glaubwürdigkeit, die Therapeut:innen ihren Patient:innen zuschreiben, und den fehlenden Ermittlungsergebnissen vonseiten der Strafverfolgungsbehörden. Das Aufdecken des Täternetzwerks „Elysium“ zeige, dass große Netzwerke nicht dauerhaft unerkannt bleiben könnten. Die Zweifel an der von ihm sog. „Rituellen-Gewalt-These“ [14, S. 249] stellten nicht infrage, dass Kinder sexualisierte Gewalt erfahren, so auch in organisiertem Kontext, und es dadurch zu einem traumatisch bedingten dissoziativen Identitätserleben kommen könne. Ähnlich beschreibt Liebrand [19] Zweifel am sog. „mind control“. Es gebe keine wissenschaftlichen Belege für die Existenz und Möglichkeit dieser „Methode“. Weiterhin legt Liebrand [19] neben dem Fallbeispiel einer Person, die von Suggestion ritueller Gewalterinnerungen durch Fachpersonen und Austausch in Onlineforen betroffen sei, mögliche psychologische Phänomene dar, die erklären, warum Menschen für solche Erklärungen suggestibel sein können. Sie nennt dabei u. a. die Suche (unspezifisch) psychisch erkrankter Menschen nach den Ursachen ihrer Leiden, den Wunsch nach Zugehörigkeit, der durch die Identität einer „Überlebenden ritueller Gewalt“ gestillt werden könne, Gruppendruck, der nach Übernahme einer solchen Identität in Betroffenenkreisen entstehe, sowie das Bedürfnis nach Einzigartigkeit und Besonderheit, dem durch besondere Aufmerksamkeit einer Fachperson nachgekommen werden könne.

## Notwendigkeit von Evidenzbasierung und Behandlungsleitlinien

Auffällige Häufungen bestimmter Diagnosen, die ansonsten sehr selten sind, können einerseits auf eine klinische Spezialisierung und damit eine gerichtete Nachfrage von Patient:innen hinweisen. Sie können aber auch der Ausdruck von Blasen sein, in denen nicht evidenzbasierte Behandlungsmethoden und Annahmen das therapeutische Milieu, z. B. einer Station, bestimmen. Es ist die Aufgabe u. a. der medizinischen Dienste der Krankenkassen, die Einhaltung therapeutischer Standards zu überprüfen. Deshalb haben sie auch Zugang zu entsprechenden Kassen- und Behandlungsdaten.

## Ausblick

In der Zukunft müssen einzelne Elemente des Themas genauer und trennschärfer benannt und hinterfragt werden. Wenn tatsächlich erlebte schwerste Gewalterfahrungen u. a. in therapeutischen, beraterischen oder Selbsthilfesettings mit verschwörungsnarrativen Elementen vermischt werden, führt das dazu, dass Betroffene Dritten gegenüber vor der Existenz- und Glaubensfrage stehen. Das heißt, wenn jemand von schwersten Gewalterfahrungen berichtet, muss darauf empathisch eingegangen werden. Beinhaltet der Erfahrungsbericht jedoch merkwürdig erscheinende und nicht belegbare Elemente, wie weitreichende kultartige Täter:innennetzwerke im Untergrund und umfassende Gedanken- und Verhaltenskontrolle, dann ist in besonderer Weise zu fordern, dass sich die Therapie in einer an der Symptomatik der Patient:innen orientierten Weise zu deren Nutzen an Plausibilitäten und realitätsnahen Überlegungen und der Abwesenheit von Belegen ausrichtet.

Wichtig erscheint uns, dass wir uns als Therapeut:innen nicht Lagern zuordnen lassen, sondern unvoreingenommen uns Anvertrautes aufnehmen. Wenn dabei Dinge berichtet werden von einer Verschwörung, einer mächtigen Elite und deren obskurer Praktiken, sollte die psychiatrische Anamnese sich auch intensiv mit dem Medienkonsum und der Infiltration über soziale Netzwerke beschäftigen.

Es existiert eine große Zahl an Betroffenenberichten und aufgedeckten Taten schwerster Formen der Gewalt, in denen manipulative und instrumentalisierte Strategien von Täterschaften beschrieben werden. Kommt die spezielle Täter:innenstrategie der scheinbar ideologischen Einbettung hinzu, können zwar Glaubenselemente zur ideologischen Rechtfertigung also zur instrumentalisiert ideologischen Begründung genutzt werden. Es handelt sich aber bei der verübten Gewalt nicht um ein Glaubensritual wie bei angeblich satanistischen Handlungen sog. ritueller Gewalt. Machtmissbrauch als Täterstrategie ist bei allen Arten sexuellen Missbrauchs zu sehen und u. E. ist eine solche ideologische Rahmung als Rationalisierung oder scheinbar ideologische Begründung des Machtmissbrauchs einzuordnen. Auch wenn die Täterschaft einer tatsächlichen Ideologie folgt, besteht die Primärmotivation nicht in der Ideologie, sondern im Rahmen der Ideologie werden Sexualstraftaten aus den bekannten Motiven von Macht, sexuellen und finanziellen Interessen verübt, wie z. B. in Sektenkontexten der Colonia Dignidad. Der Begriff rituelle Gewalt ist somit zu hinterfragen, ebenso seine „Sonderstellung“.

Dabei soll nochmals dezidiert hervorgehoben werden, dass nicht angezweifelt wird, dass es schwerste Formen der organisierten sexualisierten Gewalt ebenso wie manipulative Beeinflussung gibt [6, 8, 11]. Aber für die Art und das Ausmaß der im Zuge ritueller Gewalt beschriebenen Beeinflussung und spezifische Geheimtechniken zur Kontrolle von Persönlichkeitsanteilen gibt es keinen Beleg. Demgegenüber sind Fälle dokumentiert, in denen Pseudoerinnerungen durch entgegen den Regeln der Kunst durchgeführte Therapien oder durch den Austausch in entsprechenden Foren entstanden sind und teils der individuellen Verarbeitung von Verschwörungserzählungen entspringen.

Wenn bestimmte Erzählungen ungewöhnliche Details wiedergeben, ist dies allein kein Grund für Zweifel. Unerwartbare Details stellen im Allgemeinen eher ein Merkmal für den Realitätsgehalt von Aussagen dar. Ganz klar ist aber, dass die Entstehungsgeschichte einer Aussage und die gesamthafte, realitätsorientierte Einordnung der berichteten Inhalte zentral auch für ihre klinische Interpretation ist. Hier gilt es, gesunden Menschenverstand und Augenmaß zu bewahren. Polarisierte Lagerkämpfe und Festlegungen wie „wir glauben Betroffenen immer alles“ oder „wir gehen zunächst davon aus, die Aussage ist unwahr“ können höchstens ideologische Scheinsicherheiten produzieren, helfen im Einzelfall aber nicht weiter und erhöhen Zweifel und das Stigma in Bezug auf Betroffene und auf Therapien.

## Footnotes

1 Aus einem Obergerichtsurteil des Kantons Solothurn, Schweiz, geht folgendes hervor: „5.1 Die ‚Rituelle Gewalt‘-Theorie verbreitete sich, weil plötzlich viele Betroffene erzählten, sich daran erinnern zu können, von satanistischen Täterkreisen missbraucht worden zu sein. Beweise für solche Zirkel und Rituale, von deren Existenz auch die Beschwerdeführerin ausgeht, konnten trotz intensiver polizeilicher und staatsanwaltlicher Ermittlungen in zahlreichen Ländern nie erbracht werden. Gestützt wird die These allein durch die Berichte von Betroffenen, deren Erinnerung an schwerste psychische, physische und sexuelle Grausamkeiten meist erst später wiedererlangt wurde. Dies wird dadurch begründet, dass die Erinnerungen verdrängt worden seien und nun ‚wiederhergestellt‘ werden müssten. (…) Wenn die Betroffenen sich zuerst an keinen Missbrauch erinnern und die Erinnerungen – so auch vorliegend – erst nach und nach und in einer zunehmenden Intensivität zurückkehren, liegt der Schluss nahe, dass durch fremd- und autosuggestive Prozesse verfälschte Erinnerungen entstanden sind. Von dem geht auch die Gutachterin aus. (…) 5.2 (…) Wenn man etwas glaubt, wofür es keinen Beleg gibt, dann entzieht man sich der Realitätskontrolle. Damit lässt sich eine Theorie wie die der rituellen Gewalt hermetisch gegen Kritik abschirmen. Am Anfang steht der feste Glaube: Die Opfer werden so geschickt ferngesteuert, dass nichts ans Tageslicht kommt. Dann wird die Tatsache, dass man – wie vorliegend – nichts beweisen kann, zu einem Beleg für die Theorie. Das ist ein geschlossener Kreis.“ Abgerufen am 28.06.2023 unter: https://gerichtsentscheide.so.ch/cgi-bin/nph-omniscgi.exe?OmnisPlatform=WINDOWS&WebServerUrl=&WebServerScript=/cgi-bin/nph-omniscgi.exe&OmnisLibrary=JURISWEB&OmnisClass=rtFindinfoWebHtmlService&OmnisServer=7001&Parametername=WEB&Schema=JGWEB&Source=&Aufruf=getMarkupDocument&cSprache=DE&nF30_KEY=164094&W10_KEY=7001622&nTrefferzeile=1&Template=/simple/search_result_document.html.

2 https://icd.who.int/browse11/l-m/en#/http%3a%2f%2fid.who.int%2ficd%2fentity%2f1829103493.

3 www.clienia.ch/wp-content/uploads/Clienia-Littenheid_Bericht-ueber-externe-Untersuchung-Traumatherapie_230620.pdf.

4 Mit Datum 08.12.2022 wurde eine Patientin mit Strafbefehl wegen Falschbeschuldigung verurteilt. Bei ihr waren eine DIS diagnostiziert und im Rahmen einer Traumatherapie nachweislich unzutreffende Erinnerungen an vermeintlich schwere und rituell motivierte Missbrauchshandlungen durch die Eltern induziert/verstärkt worden. Die daraus resultierenden Strafermittlungen waren eingestellt worden. Die fortgesetzten, therapeutisch unterstützen Falschbeschuldigungen der Eltern führten zur Verurteilung der Tochter. Strafbefehl der Staatsanwaltschaft Kreuzlingen AZ SUV_K.2022.191.
